# Multiple Irradiation Affects Cellular and Extracellular Components of the Mouse Brain Tissue and Adhesion and Proliferation of Glioblastoma Cells in Experimental System In Vivo

**DOI:** 10.3390/ijms222413350

**Published:** 2021-12-12

**Authors:** Maxim O. Politko, Alexandra Y. Tsidulko, Oxana A. Pashkovskaya, Konstantin E. Kuper, Anastasia V. Suhovskih, Galina M. Kazanskaya, Lyubov S. Klyushova, Dmitry K. Sokolov, Alexander M. Volkov, Evgenii E. Kliver, Alexander A. Zheravin, Svetlana V. Aidagulova, Elvira V. Grigorieva

**Affiliations:** 1 Institute of Molecular Biology and Biophysics, FRC FTM, 630060 Novosibirsk, Russia; politko.nsu@gmail.com (M.O.P.); alexandra.tsidulko@gmail.com (A.Y.T.); anastasia-suhovskih@mail.ru (A.V.S.); Kazanskaya10101958@gmail.com (G.M.K.); klyushovals@mail.ru (L.S.K.); dmit_s95@mail.ru (D.K.S.); a_sv@ngs.ru (S.V.A.); 2 E.N. Meshalkin National Medical Research Center, 630055 Novosibirsk, Russia; o_pashkovskaja@meshalkin.ru (O.A.P.); a_volkov@meshalkin.ru (A.M.V.); kliver_68@mail.ru (E.E.K.); a_zheravin@meshalkin.ru (A.A.Z.); 3 Budker Institute of Nuclear Physics SB RAS, 630090 Novosibirsk, Russia; k.e.kuper72@gmail.com; 4 Department of Cell Biology, Novosibirsk State Medical University, 630091 Novosibirsk, Russia

**Keywords:** glioblastoma, radiotherapy, tumor microenvironment, extracellular matrix, glycosylation, mouse brain irradiation, proteoglycan expression, chondroitin sulfate, heparan sulfate, glial cell proliferation

## Abstract

Intensive adjuvant radiotherapy (RT) is a standard treatment for glioblastoma multiforme (GBM) patients; however, its effect on the normal brain tissue remains unclear. Here, we investigated the short-term effects of multiple irradiation on the cellular and extracellular glycosylated components of normal brain tissue and their functional significance. Triple irradiation (7 Gy*3 days) of C57Bl/6 mouse brain inhibited the viability, proliferation and biosynthetic activity of normal glial cells, resulting in a fast brain-zone-dependent deregulation of the expression of proteoglycans (PGs) (decorin, biglycan, versican, brevican and CD44). Complex time-point-specific (24–72 h) changes in decorin and brevican protein and chondroitin sulfate (CS) and heparan sulfate (HS) content suggested deterioration of the PGs glycosylation in irradiated brain tissue, while the transcriptional activity of HS-biosynthetic system remained unchanged. The primary glial cultures and organotypic slices from triple-irradiated brain tissue were more susceptible to GBM U87 cells’ adhesion and proliferation in co-culture systems in vitro and ex vivo. In summary, multiple irradiation affects glycosylated components of normal brain extracellular matrix (ECM) through inhibition of the functional activity of normal glial cells. The changed content and pattern of PGs and GAGs in irradiated brain tissues are accompanied by the increased adhesion and proliferation of GBM cells, suggesting a novel molecular mechanism of negative side-effects of anti-GBM radiotherapy.

## 1. Introduction

GBM remains one of the most aggressive and deadly malignant tumors [1], mainly due to its relapse, which develops in 7–9 months after the first surgery [2] and results in disease progression and patient death. Exactly these months seem to be of principal importance for the disease prognosis, and a key event at that time period is adjuvant radiochemotherapy [3]. A conventional protocol includes radiotherapy (RT) (60 Gy in 30 fractions) as an intrinsic component to eliminate the residual tumor cells [4,5]. Unfortunately, RT affects the surrounding normal brain tissue as well and develops numerous negative side-effects [6,7] with different molecular mechanisms involved [8,9]. Possibly, it interferes with the complex interrelations between GBM cells and the surrounding normal brain tissue, contributing to the formation of a specific pro-tumorous microenvironmental niche with elevated susceptibility to glioma cells’ proliferation [10,11,12,13,14]. Understanding the RT-induced changes in the tumor microenvironment (TME) and normal brain tissue may provide opportunities to target mechanisms that may promote amplified GBM aggressiveness in a previously irradiated microenvironment [15].

One of the key components of brain ECM and TME is the glycosylated molecules PGs and glycosaminoglycans (GAG) responsible for tissue structure and cell–cell and cell–matrix interaction [16]. Among PGs, chondroitin sulfate proteoglycans (CSPGs) are the main components of brain ECM, which play a special role in brain tissue organization [17,18,19]. In GBM TME, substantial amounts of CSPG are associated with non-invasive lesions, whereas low CSPG content is related to active invasion of glioma cells, suggesting CSPGs as critical regulators of brain tumor histopathology [20]. The CSPG-rich microenvironment is associated with non-invasive tumor lesions through the binding of polysaccharide CS chains with their receptor LAR (the leukocyte common antigen-related (LAR) subfamily of receptor protein tyrosine phosphatases) while the absence of CSPGs induces critical glioma invasion. The complex distribution of CSPGs in the TME can determine the nonlinear invasion behaviors of glioma cells [21]. High expression of CSPG CD44 by GBM cells is related to the mesenchymal GBM subtype and can be recommended for implementation in clinical practice for GBM subtyping [22,23,24]. Heparan sulfate proteoglycans (HSPGs) are less abundant in normal brain tissue but tightly involved in neural differentiation and glioma growth and invasion [25]. Significant increases in both polysaccharide HS content and HSPG core proteins in GBM tissues are associated with poor disease prognosis [26], supporting an important role of HS in the development of the nervous system and some of its most malignant brain tumors [27].

The effects of radiation on brain ECM and glycosylated molecules of PGs/GAGs are poorly investigated, and the recent data are summarized in the review [28]. It was shown that the radioresistance of GBM cells depends on the expression level of neuro-glial antigen 2 (NG2/CSPG4), which is the main biomarker of oligodendrocyte progenitor cells (OPCs). OPCs may induce stemness and chemo-radioresistance in GBM cells, providing a supportive function to promote the development of GBM relapse at the tumor border [29]. NG2-expressing GBM cells show resistance to ionizing radiation as well as rapidly recognizing DNA damage and effectuating cell cycle checkpoint signaling through the induction of ROS-scavenging enzymes, suggesting NG2 as an important prognostic factor for GBM patient survival [30]. Single irradiation of mouse brain with a 7 Gy dose does not affect the expression of a number of studied PGs (syndecan-1, glypican-1, HSPG2/perlecan, versican, neurocan, CD44, decorin and biglycan) but results in a significant down-regulation of brevican (3-10-fold) and NG2/CSPG4 (8-9-fold) expression in both the cerebral cortex and subcortex in a mouse experimental model [31]. From a long-term perspective, the single irradiation of normal brain with different doses (20, 30 or 40 Gy) with subsequent orthotopic implantation of nonirradiated mouse GBM tumor cells results in a more aggressive and invasive growth of the cells with the histologic features commonly observed in recurrent high-grade tumors in patients [32]. Subcurative radiation significantly increases the proportion of Ki-67-positive cells, CD44-positive cells and the number of MMP-2-positive areas in the irradiated animals, which are associated with the active proliferation, invasion, and migration of primary GBM cells [33]. Radiation-induced changes in hyaluronic acid (HA) content are related to the invasiveness of GBM cells by generating movement tracked as an ECM, and by acting as a signaling ligand for the CD44 receptor, leading to SRC activation, which is sufficient for the mesenchymal shift of GBM cells [34]. Adjuvant radiochemotherapy with temozolomide results in increased expression of glypican-1 (GPC-1), which is correlated with shorter overall survival of GBM patients [35], and affects HS content and distribution as well as causing increased intertumor heterogeneity of HPSE in relapsed tumors [36].

In previous publications, we elaborated on a mouse model to study the irradiation effect on brain tissue in vivo [37] and demonstrated short-term effects of single irradiation on the brain tissue morphology and PGs profile in mouse brain tissue [38]. However, the effects of multiple irradiation (modeling clinical RT) on brain PGs/GAGs and their functional significance remain unknown. In this study, we investigated the effects of multiple radiation on glycosylated components of the mouse brain, the molecular mechanism of their deregulation in irradiated tissue and the functional consequences of their changes for the proliferative and invasive capacity of GBM cells.

## 2. Results

### 2.1. Multiple Irradiation Affects Extracellular Components of Normal Mouse Brain Tissue

**Figure 1:**
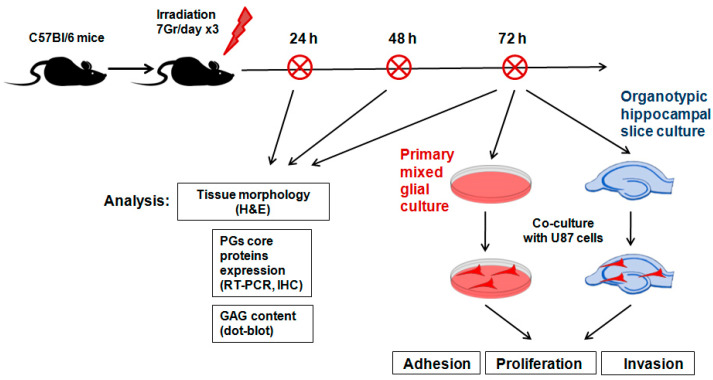
Scheme of the experiment on triple irradiation of mouse brain.

To investigate the short-term effects of multiple X-ray irradiation on the extracellular components of normal mouse brain tissue, the brains of experimental C57Bl/6 mice were irradiated using an ElektaAxesse linear clinical accelerator (6 MeV energy, three times with 24 h intervals, 7 Gy each). The scheme of the experiment is provided in Figure 1.

In total, the experimental groups included control (*n* = 6), anesthetized (*n* = 18) and irradiated (*n* = 18) animals, and their tissue morphology, expression of PG core proteins and GAG content were analyzed 24, 48 and 72 h after the last irradiation (six animals/time-point).

**Figure 2:**
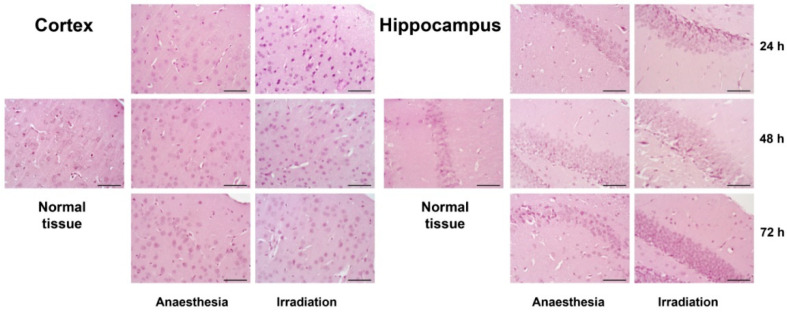
Morphology of cerebral cortex and hippocampus in the control and irradiated mouse brain tissues 24, 48 or 72 h after triple irradiation. Haematoxylin-Eosin staining. Magnification ×400, scale bar size 50 µm.

Haematoxylin-Eosin staining of the tissue samples did not reveal any significant changes in brain tissue morphology upon triple irradiation during the 24–72 h period (Figure 2).

**Figure 3:**
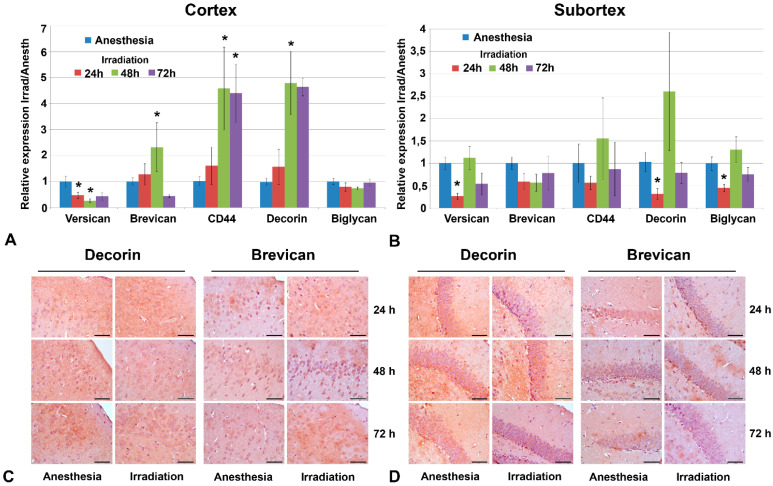
Expression levels of individual PGs in triple irradiated mouse brain tissue. (**A**,**B**) Real-time RT–PCR analysis, expression normalized to *Gapdh*, irradiated samples normalized to the appropriate control ones (anesthesia only). Bars represent the mean ± SD from triplicate experiments (OriginPro 8.5). ANOVA, a value of *p* < 0.05 was considered statistically significant (* *p* < 0.05). (**C**,**D**) Immunohistochemical staining for decorin and brevican core proteins. Magnification ×400, scale bar size 50 µm. PGs profiling and immunostaining were performed at 24, 48 and 72 h after irradiation.

However, molecular changes were observed and demonstrated a quick response to multiple irradiation in terms of the transcriptional activity of a number of CSPG core proteins (Figure 3A,B).

The observed changes were most pronounced in the cortex at 48 h after irradiation—down-regulation of versican (-2-5-fold) and up-regulation of brevican (+3-4-fold), CD44 (+6-7-fold) and decorin (+6-7-fold) with continuous restoration by 72 h after irradiation (Figure 3A). The subcortex structures demonstrated a relative resistance to irradiation in terms of PGs’ expression—down-regulation of versican (-4-fold), decorin (-3-fold) and biglycan (-2-fold) expression with a quick restoration of these changes by 72 h after irradiation (Figure 3B). Expression levels of other studied PGs proteoglycans (syndecan-1, glypican-1, HSPG2/perlecan, CSPG4/NG2 and neurocan) were not changed upon irradiation. Interestingly, an increased scatter in the data was sometimes observed in the experimental groups (in contrast to the control animals, where the scatter of data was not large). It is quite possible that the irradiation of experimental animals results in significant variability in terms of individual responses, which leads to a large scatter of the values for the studied parameters.

These early transcriptional changes were not exactly translated into the appropriate changes in the contents of the respective core proteins. Immunohistochemical staining did not reveal significant deterioration in decorin and brevican content upon irradiation in any brain zone (Figure 3C,D), although brevican demonstrated a tendency to be decreased, especially in the ECM of the subcortex structures by 72 h after irradiation (Figure 3D).

**Figure 4:**
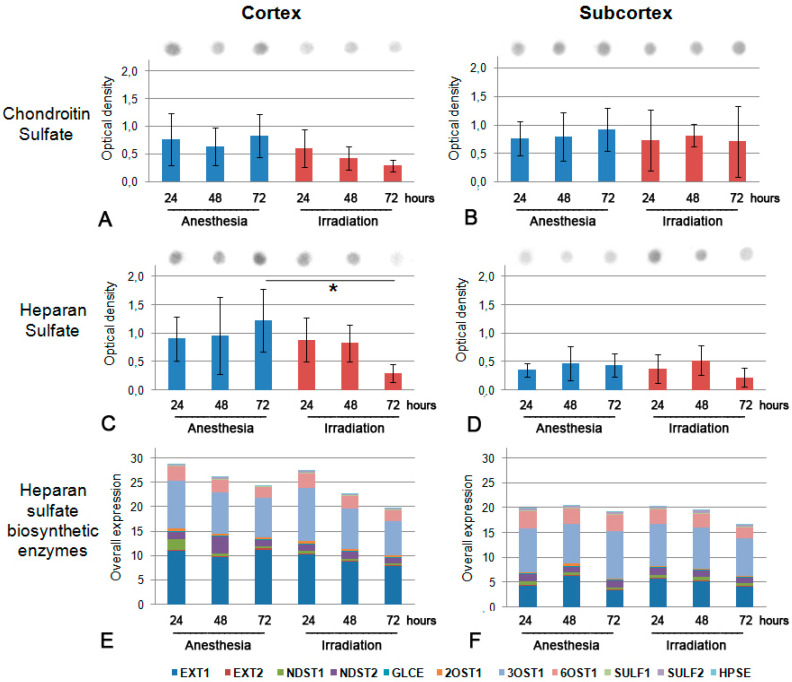
GAGs’ content in triple irradiated mouse brain tissue at 24, 48 and 72 h after irradiation. (**A**,**B**) CS content in cortex and subcortex structures (respectively). (**C**,**D**) HS content in cortex and subcortex structures (respectively). GAG content is normalized to total protein content, bars represent the mean ± SD from triplicate experiments (OriginPro 8.5). ANOVA, a value of *p* < 0.05 was considered statistically significant (* *p* < 0.05). (**E**,**F**) Overall transcriptional activity of the HS biosynthetic system in triple irradiated mouse brain tissue. The intensity of the amplified DNA fragments of HS biosynthesis-related genes was normalized to that of *Gapdh*. The stacked columns reflect the contribution of each gene to the total expression level based on the mean expression levels from triplicate experiments (Origin 8.5). Control—anesthetized animals.

As for GAG content (Figure 4A–D), the dot-blot analysis showed a 2–2.5-fold decreased overall content of HS polysaccharide chains (*p* < 0.05) and a pronounced tendency towards decreased CS content (although not statistically significant) in the cortex (Figure 4A,C) but not the subcortex (Figure 4B,D) mouse brain zones.

To look for the potential molecular mechanism of CS and HS down-regulation in irradiated brain tissues, the transcriptional activity of HS biosynthesis-related genes was investigated by real-time RT-PCR. No change in the pattern and expression levels of the studied genes (*Ext1, Ext2, Ndst1, Ndst2, Glce, Hs2st1/2Ost1, Hs3st1/3Ost1, Hs6st1/6Ost1, Sulf1, Sulf2* and *Hpse*) was detected, suggesting that the potential de-regulation of HS biosynthesis seems to occur on a post-transcriptional level (Figure 4E,F).

Overall, the obtained data demonstrate the fast reaction of PG core proteins’ expression to multiple irradiation of normal brain tissue, mainly at the transcriptional level, and a relatively fast restoration of this parameter during the observed 72 h time period. At the same time, irradiation affected the polysaccharide component of PGs and the observed decrease in the CS and HS content in irradiated tissues possessed a more pronounced effect.

### 2.2. Irradiation Inhibits the Functional Activity of Cellular Components of Normal Brain Tissue

**Figure 5:**
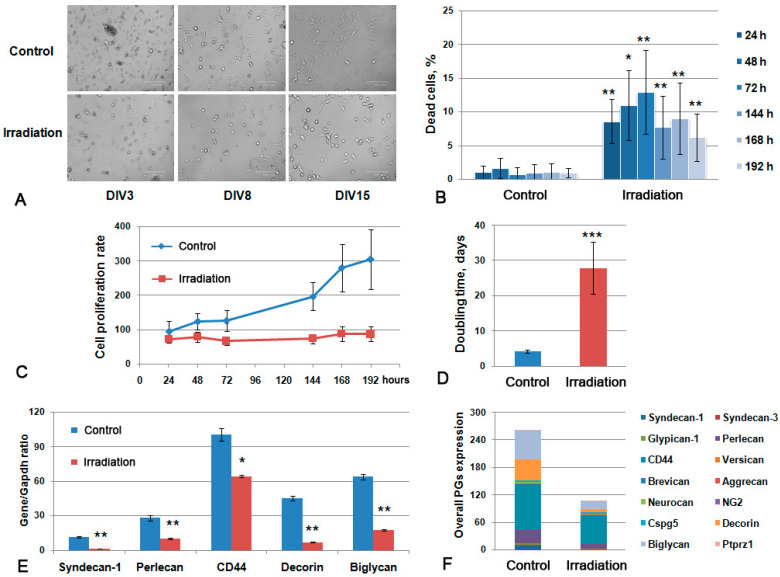
Primary glial culture from irradiated mouse brains. (**A**) Microphotograph of the obtained control and irradiated primary cultures at different Days-In-Vitro (DIV3-15). Bright-field microscopy, magnification ×200, scale bar 100 µm. (**B**) Viability of the cultures during the 24–192 h time period. (**C**) Growth curve of the primary glial cells. (**D**). Doubling time for the studied primary glial cells. Data presented on the B-D panels are obtained with the IN Cell Analyzer 2200 system. (**E**) PGs’ expression in glial primary cultures. Real-time RT–PCR analysis, intensity of the amplified DNA fragments normalized to that of *Gapdh*, bars represent the mean ± SD from triplicate experiments, ANOVA (OriginPro 8.5). (**F**) Overall transcriptional activity of PG core protein-coding genes in primary cultures from control and irradiated mouse brains. The stacked columns compare the contribution of each value to a total across categories. ANOVA, a value of *p* < 0.05 was considered statistically significant (* *p* < 0.05, ** *p* < 0.01, *** *p* < 0.001).

Brain ECM is produced mainly by glial cells, the attenuated secretory activity of which may be responsible for the observed decrease in the content of ECM components upon irradiation. To investigate the effects of multiple irradiation towards cellular components of brain tissue, primary glial cultures were established from the control and triple irradiated C57Bl/6 mouse brains (7 Gy/day) 72 h after the last irradiation using the gentleMACS™ Octo Dissociator (MiltenyiBiotec) and cultured for 20 days. The cells were monitored for proliferation rate and viability using the IN Cell Analyzer 2200 high-throughput screening platform (GE Healthcare) (Figure 5).

The primary glial cells from irradiated brains demonstrated less adhesion to poly-D-lysine-covered culture plastic and a smaller amount of cells at 15 days in vitro (DIV) (Figure 5A), lower viability (the maximal percentage of the dead cells reached 13% at 72 h of cultivation) (Figure 5B) and an extremely low proliferation rate with a doubling time of 28–29 days versus 4–5 days for normal glial primary cultures (Figure 5C,D). The observed effects suggest that the irradiation-induced inhibition of the functional activity of normal brain glial cells might result in the delayed recovery of normal brain tissue after RT.

**Figure 6:**
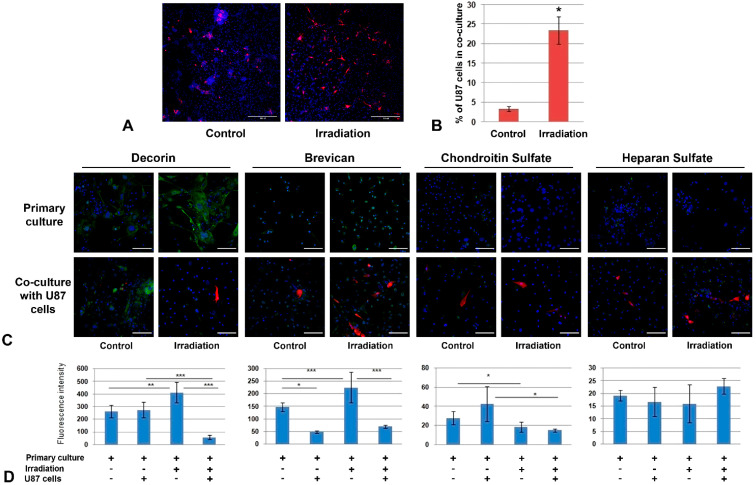
Co-culture of primary glial cultures from irradiated mouse brains with GBM cells. (**A**) Microphotograph of the co-culture of U87 GBM cells with glial cells from the control or irradiated brain tissues. Magnification ×200, scale bar 500 µm. (**B**) Quantitative analysis of a number of U87 cells per 0.5 × 0.5 cm square (Zeiss LSM710 tile-scan + CellProfiler 4.1.3). * *p* < 0.05. (**C**) Immunofluorescence analysis of PG core proteins (decorin, brevican) and GAG content (CS, HS) in primary glial cells co-cultured with U87 GBM cells or triple-irradiated or both. Visualization of the studied antigens with Alexa Fluor 488 secondary antibodies (green), U87-RFP cells (red) and DAPI (blue). Magnification ×400,scale bar 100 µm. (**D**). Quantitative analysis of the PG core proteins and GAG content in the glial cultures from control and irradiated mouse brain tissues (CellProfiler4.1.3. software). ANOVA, * *p* < 0.05, ** *p*< 0.01, *** *p* < 0.001.

Irradiation affected the capacity of the primary glial cells to express the core proteins of the main PGs and also to produce polysaccharide GAG chains (CS and HS). The overall transcriptional activity of PG core protein-coding genes in primary glial cells was significantly down-regulated (Figure 6F), mainly due to the decreased expression of predominant brain tissue PGs—syndecan-1 (-7.9-fold), perlecan (-2.8-fold), decorin (-6.5-fold), biglycan (-3.6-fold) and CD44 (-1.4-fold) (Figure 6E).

Thus, multiple irradiation of the brain results in a significant decrease in the viability and proliferative activity of glial cells in normal mouse brain tissue and their ability to produce major components of brain ECM as PGs and GAGs.

### 2.3. Irradiation-Induced Changes in PGs’ Expression and GAG Content Affect the Interaction of Primary Glial Culture with GBM Cells

To investigate the functional role of the demonstrated changes in the cellular components of normal brain tissue for GBM progression, the primary glial cultures from normal and triple-irradiated mouse brains were co-cultured with human U87 GBM cells (Figure 6).

The analysis of the adhesive and proliferative capacity of GBM cells by confocal microscopy showed a significant increase in the number of U87 cells on irradiated glial cultures (Figure 6A,B), indicating irradiated brain cells as being a more favorable substrate for cancer cells. The interaction of U87 cells with control and irradiated primary glial cultures occurred in different ways—normal glial cells enveloped GBM cells, restricting their adhesion and proliferation, whereas glial cells from irradiated brain seemed not to possess this capability (Figure 6A).

The effect was accompanied by complex changes in the PG core proteins and GAG (CS, HS) content. In spite of the decrease in mRNA levels for key brain PGs in irradiated brain tissue (Figure 5E,F), the protein content of decorin and brevican in primary glial cells was increased (+1,5-fold) but the CS content was decreased 1,5-fold, which presupposes a deterioration of the PG core proteins’ glycosylation in brain tissue upon irradiation (Figure 6C,D).

In this experiment, two variables (irradiation and presence of U87 GBM cells) developed a complex effect on PG content—decorin expression was increased upon irradiation and unchanged upon the addition of U87 cells, but the combined impact was a significant (5-8-fold) decrease in decorin content. At the same time, irradiation and U87 cells had opposite effects on brevican (+1,5-fold and -3-fold, respectively) and CS content (-1-5-fold and +1,5-fold, respectively) but their combination demonstrated the predominant effect of irradiation on CS content and those of U87 for brevican expression (Figure 6C,D). HS content in primary glial cells was more stable and did not show any significant changes upon irradiation or U87 co-culture (Figure 6C,D).

Overall, the obtained results demonstrate a significant increase in the ability of U87 GBM cells to adhere and proliferate on the primary glial cultures obtained in vitro from the triple-irradiated mouse brain compared to the control ones. Irradiation and co-culture with U87 cells resulted in complex deterioration of decorin and brevican core proteins’ expression and the contents of the polysaccharide chains of CS but not HS.

### 2.4. GBM Cells Proliferate More Actively on Organotypic Slices from Irradiated Brain Tissue

**Figure 7:**
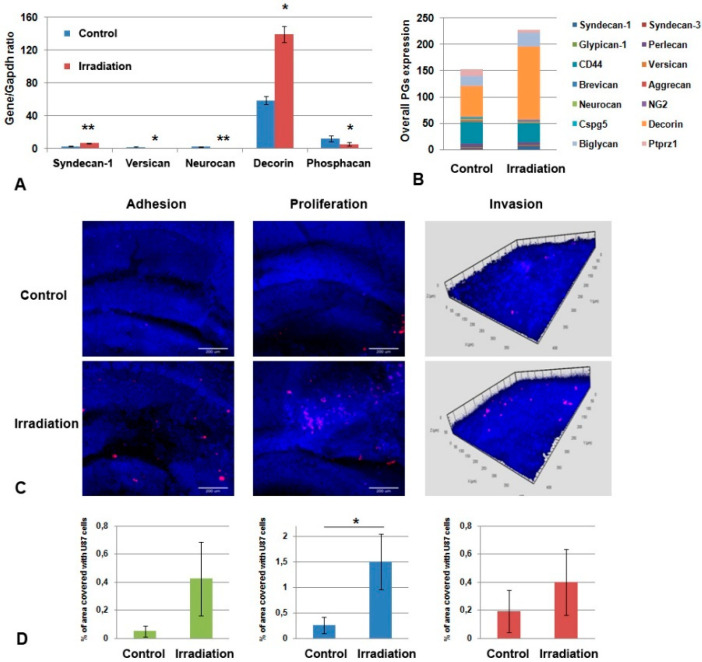
Co-culture of organotypic brain tissue slices ex vivo with GBM cells. (**A**) Effects of multiple irradiation on PGs’ expression in brain tissue. Real-time RT–PCR analysis, intensity of the amplified DNA fragments normalized to that of *Gapdh*, bars represent the mean ± SD from triplicate experiments, Student t-test (OriginPro 8.5). (**B**) Overall transcriptional activity of PG core protein-coding genes in brain tissue from control and irradiated mouse brains. The stacked columns compare the contribution of each value to a total across categories. (**C**) Confocal microscopy of U87-RFP cells seeded on the control and irradiated brain slices. Cells nuclei are stained with DAPI. Scale bar 500 μm. (**D**) Quantitative analysis of the U87-RFP cells on the control and irradiated brain tissues. ANOVA and post-hoc Fisher’s LSD test, * *p* < 0.05, ** *p* < 0.01.

To investigate the functional contribution of ECM to the irradiation effects on brain tissue, organotypic brain slices were obtained from control and irradiated brains (experimental system with preserved ECM called ex vivo [39]), characterized for PGs’ expression and co-cultured with U87 cells. The irradiated organotypic brain slices were characterized by a significant increase in the mRNA levels of some PG core proteins (syndecan-1 and decorin, 2-fold) (Figure 7A) and elevated overall transcriptional activity of the main PGs (Figure 7B).

Co-culture of the irradiated brain slices with GBM U87 cells revealed a significant increase in the adhesion and proliferative activity of the cancer cells on organotypic brain slices ex vivo (* *p* < 0.05) and a tendency towards a more active invasion of U87 cells within the brain slices (Figure 7C,D). These results suggest that intact brain tissue is capable of restricting the adhesion and proliferation of GBM cells, whereas irradiated tissue loses this ability.

Taken together, the obtained data, for the first time, demonstrate the proof-of-principle that the irradiation-induced changes in glycosylated components (PGs, GAGs) of normal brain tissue might contribute to the adhesion and proliferation of GBM cells on irradiated brain tissue and GBM relapse development.

## 3. Discussion

The obtained results regarding the negative effects of multiple irradiation on PGs’ expression and GAGs’ composition in normal mouse brain tissue support and extend our previous data on the ability of even a single 7 Gy irradiation to interfere in the transcriptional activity of some PG-coding genes [31]. This dose was chosen from the literature data and validated further in terms of the absence of evident effects on brain tissue morphology in a single-irradiation study. In fact, the choice of radiation dose in the development of each specific experimental model is very important and the literature describes a variety of radiation dosages used—1 Gy [40], 2 Gy [41], 4 Gy [42,43,44], 5 Gy [45,46], 7 Gy [47,48,49], 8 Gy [41,50], 10 Gy [51,52,53,54], 20 Gy [41,55], 25 Gy [54,56] and 30 Gy [57] depending on the study purpose and design.

This study represents a complex investigation in which analysis of the expression levels of a comprehensive set of PGs (syndecan-1, glypican-1, HSPG2/perlecan, versican, neurocan, brevican, NG2/CSPG4, CD44, decorin and biglycan) upon multiple irradiation was performed for the first time. For the listed PGs, only NG2/CSPG4 [29,30], CD44 [33] and glypican-1 [35] were identified previously as radiation-related genes. Irradiation-induced changes in decorin and brevican expression, for the first time, revealed them as radiation-responsive genes involved in RT side-effects.

Another important RT-induced change in brain tissue was a significant brain-zone-dependent (in the cortex but not the subcortex) decrease in the contents of CS and HS, which play an important functional role in brain tissue organization and GBM development along with numerous other radiation-responsive genes [58,59,60,61] and miRNA [62]. It was shown that CSPGs, being a major component of the brain ECM, serve as a central organizer of the brain TME, contribute to its physical structure and induce or inhibit glioma invasion by regulating the dynamics of the CSPG receptor LAR as well as the spatiotemporal activation status of resident astrocytes and tumor-associated microglia [21]. The fundamental switch between two distinct states of GBM (invasion and non-invasion) is directly associated with the amount of glycosylated CSPGs in the tumor ECM, which induce the activation of tumor-associated microglia and the formation of the astrogliotic capsule that directly inhibits tumor invasion. Its absence from GBM presents an environment that is favorable to diffuse infiltration and GBM progression [20]. Previously, we showed that the degradation of CS in brain ECM directly affects the adhesion and invasion of GBM cells to brain tissue [38]. The results presented here demonstrate that this important component of brain tissue is responsive to multiple X-ray irradiation, which results in a decrease in CSPG expression and CS content in normal brain tissue and activated adhesion and proliferation of GBM cells in irradiated brain tissue.

Interestingly, no significant changes were detected in HSPGs’ expression upon triple irradiation, although the published data demonstrate an involvement of HSPGs, polysaccharide HS molecules and their degrading enzyme heparanase (HPSE) in the migration and invasion of cancer cells and glioma invasion and progression [63,64,65]. It turns out that different PGs/GAGs respond differently to multiple irradiation—some CSPG core proteins/CS respond to it, while HSPGs show resistance.

Since the main producer of PGs/GAGs in the brain tissue are glial cells, a comparative analysis of glial primary cultures from the control and triple-irradiated brain tissues was performed in this study. Multiple irradiation resulted in a significant decrease in the viability and proliferative activity of the glial cells, affecting their ability to produce major components of brain ECM as PGs/GAGs and resist the adhesion and proliferation of GBM U87 cells in co-culture systems in vitro and ex vivo. These data remain consistent with previously published results on the role of microglia in GBM progression. It was shown in the literature that glial cells (comprising ~50% of all brain cells) are tightly involved in GBM cells’ growth and limited response to radiation or chemotherapy [66]. Microglial cells (MG) affect the migration and proliferation of GBM cells in 2D and 3D co-culture systems, eliciting drug resistance to chemotherapeutic drugs (especially in 3D cultures) [67]. Glioma-associated microglia and macrophages (GAMs) contribute to GBM biology, with direct involvement in the degradation of the ECM through GAM-secreted factors, which represents a crucial mechanism that allows the expansion of tumors and parenchyma invasion [68]. The results presented here show for the first time that glial cells might contribute to the development of GBM not only through the degradation of the ECM but also through a decrease in the production of its key components.

Our data on the involvement of normal glial cells in the adhesion and proliferation of GBM cells through the reorganization of glycosylated components of brain ECM suppose a novel molecular mechanism underpinning the negative effects of RT on GBM cells’ survival and on relapse development—irradiation/inhibition of the biosynthetic activity of glial cells/deterioration of the PG expression and GAG content/reorganization of brain ECM/proliferation and invasion of the residual glioma cells/GBM relapse development.

To prevent or attenuate the negative consequences of the multiple irradiation to normal brain tissue, it may be worth trying to revise the single dose of radiation (downward), similarly to how it was applied in the case of salvage RT in the treatment of recurrent GBM [69]. The identification of decorin, brevican and CS/HS as key components of the brain tissue that respond to multiple irradiation would outline the direction of their further study as potential markers of the radiotoxicity of the chosen RT regimen on normal brain tissue or as targets for the development of new protective drugs to protect brain tissue.

## 4. Materials and Methods

### 4.1. Animals and Tissue Samples

All experiments were performed on 2-month-old male C57BL/6 mice (*n* = 54) obtained from the SPF animal house at the Institute of Cytology and Genetics (Novosibirsk, Russia). Animals were housed in polycarbonate cages at a temperature of 25 ± 1 °C, a humidity of 50–60%, under 12/12 h light/dark cycles, with free access to water and food. Animals were sacrificed by cervical dislocation (AVMA Guidelines for the Euthanasia of Animals, 2013). Freshly excised brain tissue was divided, and half of the tissue was immediately fixed in 10% neutral buffered formalin, then routinely processed and embedded in paraffin using the Shandon Citadel 2000 Tissue Processor and the HistoStar Embedding Workstation (Thermo Fisher Scientific, Waltham, MA, USA). The second half was divided into cortex and subcortex sections and was preserved with RNALater solution (Thermo Fisher Scientific, Waltham, MA, USA) according to the manufacturer’s instructions. All procedures with the experimental animals were conducted in accordance with the European Communities Council Directive 2010/63/EU and approved by the local Committee for Biomedical Ethics of the Institute of Molecular Biology and Biophysics FRC FTM (code 07-11012017). All efforts were made to minimize animal suffering and to reduce the number of animals used.

### 4.2. Animals’ Irradiation

The detailed protocol for animal irradiation and the justification for the irradiation parameters chosen are described in [31,37]. Briefly, to anesthetize mice, intraperitoneal injection with Domitor (Orion Corporation, Espoo, Finland; 0.125 mg/kg of body weight) followed by Zoletil injection after 10 min (Valdepharm, Valdreuil, France; 20 mg/kg) into another part of mouse abdomen was used. The anesthetized animals were irradiated with a single dose of 7 Gy during three consecutive days (total dose of 21 Gy) using the ElektaAxesse clinical linear accelerator (Elekta Ltd., Crawley, UK) (E.N. Meshalkin National Medical Research Center, Novosibirsk, Russia) or the VEPP-4 research accelerator complex (Budker Institute of Nuclear Physics SB RAS, Novosibirsk, Russia). Brain tissue samples were obtained after 24 h, 48 h or 72 h after the last irradiation (6 animals/each group).

Irradiation zones included the whole mouse brain (for ElektaAxesse) or the whole mouse head (for VEPP-4), and irradiation doses were given according to the X-ray sources settings and controlled using Gafchromic EBT3 radiochrome film (Ashland Specialty Ingredients, Wilmington, DE, USA) according to Politko et al. [37]. The control irradiated film was scanned with a Canon 9000f mark II (Canon, Tokyo, Japan) and quantification analysis was performed using the ImagePro6 program.

### 4.3. Real-Time RT-PCR Analysis

Total RNA was extracted from the tissue samples using the QIAzol Lysis Reagent (Qiagen, Germantown, MD, USA) according to the manufacturer’s instructions. The integrity and quality of the isolated RNA were checked by agarose gel electrophoresis, and total RNA concentration was measured with the Qubit–iT RNA Assays Kit (Thermo Fisher Scientific, Waltham, MA, USA) according to the manufacturer’s instructions. cDNA was synthesized from 0.5μg of total RNA using a First Strand cDNA Synthesis kit (Fermentas, Hanover, MD, USA) and 1/10th of the product was subjected to PCR analysis. Real-time PCR for mouse PGs and HS metabolic enzymes was performed using the CFX96 Real-Time PCR Detection System (Bio-Rad, Hercules, CA, USA) and the Taq-polymerase (IMCB, Novosibirsk, Russia), SYBR Green (Thermo Fisher Scientific, Waltham, MA, USA). The PCR conditions were: 95 °C for 3 min, then 95 °C for 20 s, 59 °C for 15 s, and 72 °C for 30 s for 40 cycles; the total reaction volume was 25 μL. *Gapdh* was used as the reference gene. The fold change for each mRNA was calculated using the 2^−ΔCt^ method. Six biological replicates were used for RT-PCR analysis. The used primers are listed in Table 1.

### 4.4. Histological Study

Routine staining of 3.5-μm coronal tissue sections with Haematoxylin and Eosin (H&E) was performed on a Microm HMS740 (Thermo Scientific, Waltham, MA, USA) and documented by light microscopy (Axiostar Plus, Zeiss, Jena, Germany) with magnification ×400. Ten microscopic fields were estimated for each specimen; the staining was analyzed independently by two qualified pathologists.

### 4.5. Immunohistochemical Analysis

For immunohistochemistry, 3- to 4-µm sections of formalin-fixed, paraffin-embedded tissue were deparaffinized, and antigen was retrieved by treatment with sodium citrate buffer (10 mM sodium citrate, 0.05% Tween-20) at 95–98 °C for 20 min. Non-specific binding blocking, immunostaining and visualization were performed using Mouse- and Rabbit-specific HRP/DAB (ABC) Detection IHC kits (Abcam cat. N ab64264) according to the manufacturer’s instructions. The rabbit polyclonal anti-decorin (Abcam, cat. N ab175404, 1:100) and rabbit polyclonal anti-brevican antibody (Abcam cat. N ab111719, 1:100) primary antibodies were used for immunostaining for 1 h at room temperature. The sections were counterstained with hematoxylin and observed by light microscopy using an AxioScopeA1 microscope (Zeiss, Jena, Germany). Quantitative analysis was performed with the ZENblue program (Zeiss, Jena, Germany).

### 4.6. Immunocytochemical Analysis

For ICC experiments, cells were grown on coverslips coated with poly-D-lysine (Gibco, Waltham, MA, USA). Cells were washed once with PBS prior to fixation with 4% PBS-buffered formaldehyde (10 min, RT). After 3 × PBS rinse, cells were permeabilized with 0.1% Triton-X100 in PBS for 5 min and washed three times with PBS. Non-specific antibody binding was blocked with PBST (PBS with 0.05% Tween-20) containing 1% BSA (Sigma-Aldrich, St. Louis, MO, USA) and the samples were incubated with primary antibodies diluted in 1% BSA in PBST for 1 h at room temperature: rabbit anti-decorin antibody (Abcam ab175404, 1:100), rabbit anti-brevican antibody (Abcam ab111719, 1:100), mouse anti-CS antibody (Sigma-Aldrich C8035, CS-56, 1:100) or mouse anti-HS antibody (Millipore, clone T320.11, 1:100). Following 3 × 5 min washes with PBST, the samples were incubated for 1 h in the dark with anti-mouse or anti-rabbit AlexaFluor-488-labelled secondary antibodies (Abcam ab150117 or ab150077, respectively, 1:1000). Samples were washed three times with PBST, and mounted on slides using SlowFade Diamond mounting medium with DAPI (Thermo Scientific, Waltham, MA, USA). Slides were analyzed with an LSM 710 laser confocal microscope (Zeiss, Jena, Germany).

### 4.7. Dot-Blots for Total GAG Content

Brain tissue samples were lysed with RIPA-buffer (Thermo Scientific, Waltham, MA, USA) containing “Complete” Protease Inhibitor Cocktail (Roche, Indianapolis, IN, USA), sonicated using a Microson XL-2000 Ultrasonic liquid processor (Qsonica, Newtown, CT, USA) at 15 µm amplitude (45 W) and centrifuged for 15 min at 14,000 gat + 4 °C. The protein concentration was quantified using the Pierce™ BCA Protein Assay Kit (Thermo Scientific, Waltham, MA, USA). Quantities of 1 μg of total proteins were dot-blotted onto PVDF membranes (Millipore, Burlington, MA, USA in a volume of 1μL. The membranes were blocked with 5% non-fat milk in PBST for 1 h and incubated with mouse anti-CS primary antibody (Sigma-Aldrich C8035, CS-56, 1:500) or anti-HS antibody (Millipore, clone T320.11, 1:500) diluted in 1% non-fat milk in PBST overnight at 4 °C followed by the secondary peroxidase-conjugated antibody goat anti-Mouse IgG (Abcam, Cambridge, UK) for 1 h at room temperature. GAGs were detected with an Optiblot ECL Detection Kit (Abcam, Cambridge, UK) according to the manufacturer’s instructions. Blots were imaged using ChemiDoc (BioRad, Hercules, CA, USA) and analyzed semi-quantitatively using ImageLab 6.0.1 (BioRad, Hercules, CA, USA) software, the chemiluminescent signal was normalized to total protein content according to Coomassie staining.

### 4.8. Primary Culture

Primary mixed glia was obtained using the gentleMACS™ Octo Dissociator with Heaters, and the Adult Brain Dissociation Kit (MiltenyiBiotec, Bergisch Gladbach, Germany), according to the manufacturer’s protocol. Briefly, two 10-week-old C57BL/6J mice per experimental group were sacrificed by cervical dislocation, and brains were removed and washed in cold PBS once. Brains were cut into 0.5 cm slices and transferred to C Tubes (MiltenyiBiotec, Bergisch Gladbach, Germany) containing enzyme mix 1. Enzyme mix 2 was added and the brains were dissociated using the gentleMACS program 37C_ABDK_01. After the termination of the program, the suspension was filtered through a 70-μm strainer and centrifuged at 300× *g* for 10 min at room temperature. The pellet was resuspended in Debris Removal Solution and debris was removed by centrifugation at 3000× *g* for 10 min at room temperature. Cells were washed with cold PBS, incubated with 1 × Red Blood Cell Removal Solution for 10 min at 4 °C and centrifuged at 300× *g* for 10 min at room temperature. The pelleted cells were resuspended in IMDM (Gibco, Waltham, MA, USA) medium supplemented with 2 mM L-glutamine, 100 units/mL penicillin, 100 μg/mL streptomycin and 10% fetal bovine serum. Cells were seeded onto poly-D-lysine-covered flasks and incubated at 37 °C in a humidified 5% CO_2_ incubator. The medium was changed completely after 24 h and ½ of the medium was changed every 3 days. Primary cultures were grown for 11 days (up to 90% confluency) before being passaged for further experiments: some cells were seeded onto coverslips for ICC analysis and co-culture with U87-RFP cells; some cells—onto 96-well plates for proliferation assay; some cells—into culture flasks for RT-PCR analysis. After passaging, cells were grown for 9 additional days before analyses, totaling 20 days in culture.

### 4.9. Cell Viability and Proliferation Assay

Cell viability and proliferation were detected by Hoechst 33342/propidium iodide (PI) staining using the IN Cell Analyzer 2200 (GE Healthcare, Chicago, IL, USA). Primary mixed glial cells from control and irradiated brains were seeded onto 96-well plates at 10^4^ cells per well and allowed to attach and grow for 24 h. Cells’ viability and proliferation were analyzed at 24, 48, 72, 144, 168 and 192 h after plating. Cells were stained with Hoechst 33,342 (Sigma-Aldrich, St. Louis, MO, USA) for 30 min at 37 °C and PI (Sigma-Aldrich, St. Louis, MO, USA) for 10 min at 37 °C. An IN Cell Analyzer 2200 cell imaging system (GE Healthcare, Chicago, IL, USA) was used to perform automatic imaging of six fields per well under 200× magnification, in brightfield and fluorescence channels. The images produced were used to analyze live and dead cells using the IN Cell Investigator software (GE Healthcare, Chicago, IL, USA).

### 4.10. Organotypic Hippocampal Slice Culture

Organotypic hippocampal slice cultures (OHSCs) were prepared according to the previously described protocol [70] with modifications. Briefly, control or irradiated mice were sacrificed by cervical dislocation, decapitated, and the brains were rapidly removed under aseptic conditions and placed into ice-cold Hank’s balanced solution with 0.9% glucose. The hippocampi were removed and cut rapidly into 500 μm transversal slices with a manual McIlwain tissue chopper (Stoelting Co., Wood Dale, IL, USA). The slices were transferred to Millicell culture inserts (Millipore, PICM0RG50) placed into a 6-well plate containing 1.2 mL of culture medium, consisting of Neurobasal medium supplemented with B27 and GlutaMAX (Gibco, Waltham, MA, USA). The organotypic hippocampal slices were cultivated in a 90% humidified atmosphere with 5% CO_2_ at 37 °C. The medium was changed on the next day, and from day 3 of the experiment, the medium was changed twice a week.

### 4.11. Co-Culture of GBM Cells with the Primary Brain Cultures In Vitro and Organotypic Cultures Ex Vivo

For co-culture experiments, human GBM U87 cells stably expressing RFP (U87-RFP cells) (AntiCancer Inc., San Diego, CA, USA) were used. Cells were maintained in IMDM medium supplemented with 2 mM L-glutamine, 100 units/mL penicillin, 100 μg/mL streptomycin and 10% fetal bovine serum at 37 °C in a humidified 5% CO_2_ incubator. For co-culture with U87-RFP cells, primary mixed glial cultures were grown up to 70% confluency on poly-D-lysine-coated coverslips placed in 12-well plates. In addition, 10^3^ U87-RFP cells were added to each well and co-cultivated for 7 days. Co-cultures were washed once with PBS prior to fixation with 4% PBS-buffered formaldehyde (10 min, room temperature) and used for immunostaining.

For co-culture with organotypic slices, U87-RFP cells were harvested using trypsin/EDTA, pelleted by centrifugation and resuspended in Neurobasal medium.

To analyze the adhesion of tumor cells to the surface of organotypic slices, 12,500 cells in 10 μL were applied onto each slice, incubated for 2 h at 37 °C and 5% CO_2_, washed in PBS, and fixed in 10% neutral buffered formalin at 4 °C for 16 h. To assess the proliferation of tumor cells and their invasion into organotypic slices, 5000 cells in 10 μL were applied onto each slice, incubated for 7 days at 37 °C and 5% CO_2_, then washed with PBS and fixed in 10% neutral buffered formalin at 4 °C for 16 h. After fixation, organotypic slices-U87-RFP co-cultures were washed three times in PBS, transferred onto microscope slides and covered with a coverslip using SlowFade Diamond medium with DAPI (Thermo Scientific, Waltham, MA, USA). These co-cultures were visualized using LSM 710 laser confocal microscope (Zeiss, Jena, Germany). Images were acquired in z-stack and tile-scan modes to visualize the signal over the entire volume of the slice. The acquisition, processing and analysis of images were performed using the ZEN Black 2012 software (Zeiss, Jena, Germany). Tumor cell adhesion was determined as the percentage of the slice surface area occupied by tumor cells (summarized signal from 20 µm deep slice volume); the proliferation of tumor cells was determined as a percentage of the area occupied by tumor cells in the maximum intensity projection mode (summarized signal from the whole slice volume); the invasion of tumor cells was determined as a percentage of the area occupied by tumor cells at a depth of 25 µm from the slice surface.

### 4.12. Statistical Analysis

ANOVA analysis with Fisher’s Least Significant Difference (LSD) Post-Hoc test was performed to determine statistical significance between the studied groups. Data were expressed as means ± SD. Statistical analysis was performed using ORIGIN Pro 8.5 software; a value of *p* < 0.05 was considered to indicate a statistically significant difference.

## Figures and Tables

**Table 1:** Sequences of primers used in PCR analysis.

Gene	Sequence
*Sdc1*	5′-GGTCTGGGCAGCATGAGAC-3′
	5′-GGAGGAACATTTACAGCCACA-3′
*Gpc1*	5′-CTTTAGCCTGAGCGATGTGC-3′
	5′-GGCCAAATTCTCCTCCATCT-3′
*Hspg2*	5′-CCGTGCTATGGACTTCAACG-3′
	5′-TGAGCTGTGGAGGGTGTATG-3′
*Vcan*	5′-GGAGGTCTACTTGGGGTGAG-3′
	5′-GGGTGATGAAGTTTCTGCGAG-3′
*Bcan*	5′-GTGGAGTGGCTGTGGCTC-3′
	5′-AACATAGGCAGCGGAAACC-3′
*Cspg4*	5′-TCTTACCTTGGCCCTGTTGG-3′
	5′-ACTCTGGTCAGAGCTGAGGG-3′
*Dcn*	5′-CCCCTGATATCTATGTGCCC-3′
	5′-GTTGTGTCGGGTGGAAAATC-3′
*Bgn*	5′-GCCTGACAACCTAGTCCACC-3′
	5′-CAGCAAGGTGAGTAGCCACA-3′
*Ncan*	5′-F CCAGCGACATGGGAGTAGAT-3′
	5′-GGGACACTGGGTGAGATCAA-3′
*CD44*	5′-CAAGTTTTGGTGGCACACAG-3′
	5′-AGCGGCAGGTTACATTCAAA-3′
*Gapdh*	5′-CGTCCCGTAGACAAAATGGT-3′
	5′-TTGATGGCAACAATCTCCAC-3′

## Data Availability

All data generated or analyzed during this study are included in this published article.
